# Coculture with macrophages alters ferroptosis susceptibility of triple-negative cancer cells

**DOI:** 10.1038/s41420-024-01884-w

**Published:** 2024-03-01

**Authors:** Hiroto Konishi, Yuya Haga, Moe Okumura, Hirofumi Tsujino, Kazuma Higashisaka, Yasuo Tsutsumi

**Affiliations:** 1 Graduate School of Pharmaceutical Sciences, Osaka University, 1-6 Yamadaoka, Suita, Osaka 565-0871 Japan; 2 School of Pharmaceutical Sciences, Osaka University, 1-6 Yamadaoka, Suita, Osaka 565-0871 Japan; 3 Museum Links, Osaka University, 1-13 Machikaneyama, Toyonaka, Osaka 560-0043 Japan; 4 Institute for Advanced Co-Creation Studies, Osaka University, 1-6 Yamadaoka, Suita, Osaka 565-0871 Japan; 5 Global Center for Medical Engineering and Informatics, Osaka University, 2-2 Yamadaoka, Suita, Osaka 565-0871 Japan; 6 Institute for Open and Transdisciplinary Research Initiatives, Osaka University, 1-1 Yamadaoka, Suita, Osaka 565-0871 Japan

**Keywords:** Cell death, Breast cancer

## Abstract

Various treatment options, such as molecular targeted drugs and immune checkpoint blockades, are available for patients with cancer. However, some cancer types are refractory to molecular targeted therapies or acquire drug resistance after long-term treatment. Thus, ferroptosis, a newly defined type of programmed cell death caused by the iron-dependent accumulation of lipid peroxidation, has gained attention as a novel cancer treatment strategy. Understanding cell–cell interactions in the tumor microenvironment is important for the clinical application of ferroptosis inducers. However, the effects of cell–cell interactions on ferroptosis sensitivity remain unclear. Thus, we aimed to evaluate the effects of macrophage–cancer cell interactions on ferroptosis induction. Coculture experiments showed that conditioned medium prepared from macrophages did not alter the ferroptosis sensitivity of cancer cells. By contrast, coculture via transwell, which enables cell–cell interactions through secretion, increased the sensitivity of cancer cells to ferroptosis inducers. Additionally, direct coculture increased the susceptibility of cancer cells to RSL3-induced ferroptosis. Mechanistically, coculture with macrophages upregulated the levels of intracellular ferrous ions and lipid peroxidation in cancer cells. These findings provide novel insights into the mechanisms by which cell–cell interactions influence ferroptosis induction and application of ferroptosis inducers as a cancer treatment option.

## Introduction

With the recent advancements in understanding the biological mechanisms of cancer, various drugs, such as molecular targeted drugs or immune checkpoint blockades, have been approved for cancer treatment [1, 2]. Despite these therapeutic options, several types of refractory cancer are still reported in the clinic. For instance, pancreatic cancer is a refractory cancer owing to the lack of early diagnosis and biomarkers [3] Additionally, clinical trials have failed to show the benefit of molecular targeted drugs in triple-negative breast cancer because of the heterogeneous pathology of this disease [4]. In addition, patients with cancer, such as epidermal growth factor receptor-mutated nonsmall cell lung cancer or B-Raf proto-oncogene, serine/threonine kinase (BRAF)-mutated melanoma, inevitably acquire drug resistance after long-term treatment [5, 6]. Although several attempts have been made to overcome these refractory cancers, novel drugs that can eliminate these cancers are still needed. Almost all molecular targeted drugs aim to induce apoptosis in cancer cells, and novel types of agents that specifically induce cancer cell death are urgently needed.

Ferroptosis, a novel type of cell death that was first described in 2012 [7], has attracted attention as a therapeutic approach for cancer because ferroptosis has been discovered by screening drugs specifically targeting cancer cells. This process is caused by the accumulation of lipid peroxides generated by intracellular ferrous ions.

Acyl-CoA synthetase long-chain family member 4, which determines ferroptosis sensitivity by altering cellular lipid composition, is highly expressed in triple-negative breast cancer compared with other breast cancer subtypes [8]. Additionally, artesunate, the first-line drug for malaria, inhibits the survival of sunitinib-resistant renal cell carcinoma by inducing ferroptosis [9]. However, little is known about the ferroptosis susceptibility of cancer cells in the tumor microenvironment (TME), which is the specific environment surrounding cancer cells [10]. Thus, the effects of cell–cell interactions in the TME on ferroptosis sensitivity remain largely unknown. The TME is composed of cancer cells, endothelial cells, macrophages, fibroblasts, T cells, B cells, dendritic cells, and natural killer cells [10] and has been proposed as a determinant of drug sensitivity in some cancer types. For example, hepatocyte growth factor secretion from stromal cells promotes resistance to RAF inhibitors in BRAF-mutant melanomas [11].

Understanding the mechanisms by which cancer cell–other cell type interactions affect ferroptosis induction is essential for developing ferroptosis inducers as anticancer drugs. Among several types of cell types in TME, macrophages play a crucial role in iron metabolism [12]. However, the effect of macrophage–cancer cell interaction on ferroptosis induction remains unclear. Herein, we aimed to explore the effects of the interaction between macrophages and cancer cells on ferroptosis induction in triple-negative breast cancer cells utilizing several coculture models. This study provides insight into the potential use of ferroptosis inducers as a promising cancer treatment strategy.

## Results

### Indirect interaction of cancer cells and macrophage promotes ferroptosis induction

**Fig. 1:**
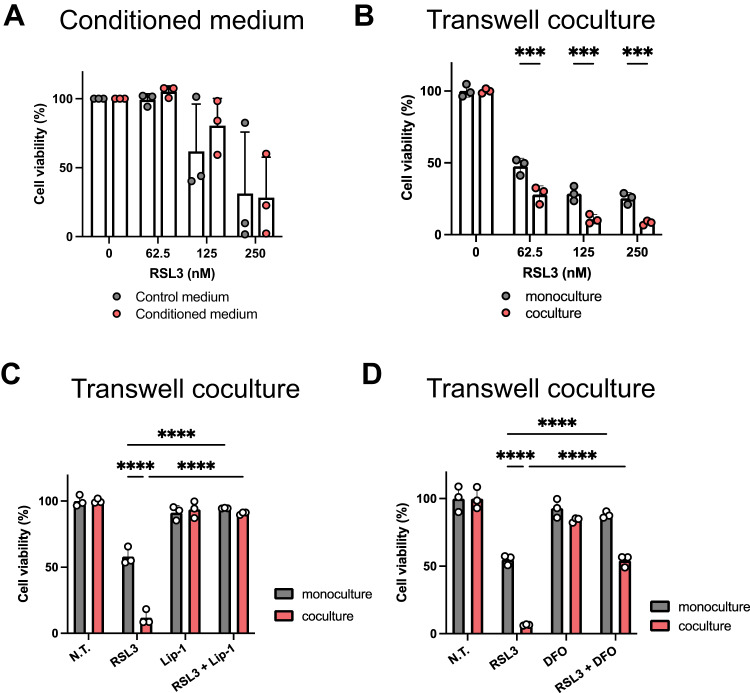
Indirect coculture of cancer cells with macrophage alters ferroptosis sensitivity. **A** 4T1-Luc cells were seeded in a 96-well plate (2000 cells/well) and then incubated with RPMI-1640 containing 10% FBS and 1% antibiotics (control medium) or conditioned medium, which was four-fold diluted from the supernatant of RAW264.7 cells for 48 h. After that, cells were incubated with the indicated concentrations of RSL3 for 24 h. Cell viability was determined using the MTT assay. Data are expressed as the means ± standard deviation (SD) of three independent experiments. **B** 4T1-Luc cells (5000 cells/well) were cultured alone or with RAW264.7 (5000 cells/well) cells in a 24-well plate and transwell insert with 0.4 µm pore size. After 72 h of incubation, the cells were treated with the indicated concentrations of RSL3 for 16 h, and cell viability was determined using the MTT assay. Data are means ± SD (*n* = 3). This experiment was repeated twice with similar results. **C** After 72 h of incubation, the cells were treated with RSL3 (125 nM), Lip-1 (1 µM), or their combination for 16 h. Cell viability was determined using the MTT assay. Data are presented as means ± SD (*n* = 3). This experiment was repeated twice with similar results. **D** After 72 h of incubation, the cells were treated with RSL3 (125 nM), DFO (5 µM), or their combination for 16 h. Cell viability was determined using the MTT assay. Data are expressed as means ± SD (*n* = 3). This experiment was repeated twice with similar results. ****P < 0.0001, ***P < 0.001 by two-way analysis of variance (ANOVA), followed by Bonferroni’s multiple comparison test. N.T. not treated.

In the present study, we utilized 4T1-Luc cells as a model of triple-negative breast cancer cell line and RAW264.7 cells as a model of macrophage cells due to their macrophage-like characteristics [13]. The supernatant from the culture of RAW264.7 cells was collected and treated with 4T1-Luc cells to clarify the effect of the conditioned medium prepared from RAW264.7 cells on the ferroptosis sensitivity of 4T1-Luc cells. The MTT assay was conducted to assess the ferroptosis induced by RSL3, a glutathione peroxidase 4 (GPx4) inhibitor. Results showed no significant difference between the groups treated with or without the conditioned medium (Fig. 1A). Subsequently, 4T1-Luc cells were cultured alone or with RAW264.7 cells using a transwell chamber to enable communication between the two cell types through their secretions. After RSL3 treatment, ferroptosis sensitivity was assessed, and cell viability was evaluated using the MTT assay. Results demonstrated that RSL3 significantly decreased the viability of 4T1-Luc cells when cocultured with RAW264.7 cells (Fig. 1B). Alternatively, we also assessed cell viability using crystal violet staining. Results demonstrated that the remaining 4T1-Luc cells were stained and that the cell area was smaller under the coculture conditions than under the monoculture conditions (Supplementary Fig. 1). To further confirm that the cell death caused by RSL3 was due to ferroptosis, we tested the effect of combination treatment with RSL3 and ferroptosis inhibitors Lip-1 and DFO. Lip-1 prevents lipid peroxidation and inhibits ferroptosis [14], whereas DFO inhibits ferroptosis by acting as an iron chelator [15]. MTT assay results demonstrated that the combination of RSL3 with either Lip-1 or DFO significantly decreased cell death caused by RSL3 treatment under both monoculture and coculture conditions (Fig. 1C, D). Taken together, these results might suggest that indirect coculture with macrophages can increase ferroptosis sensitivity in triple-negative breast cancer cells.

### Direct coculture of cancer cells and macrophages promotes ferroptosis

**Fig. 2:**
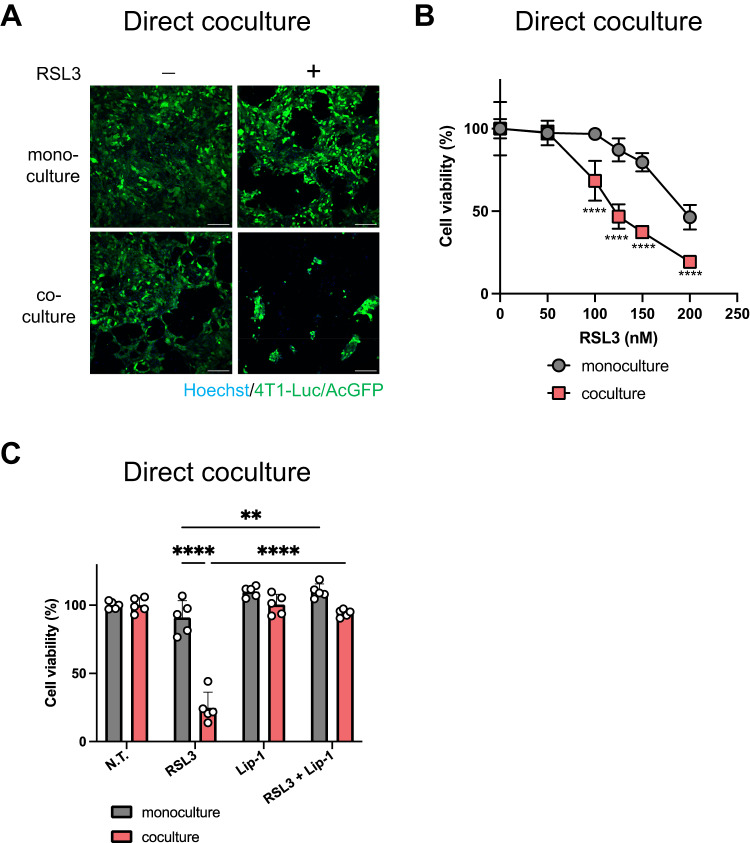
Effect of direct coculture of cancer cells with macrophages on ferroptosis sensitivity. **A** 4T1-Luc/AcGFP cells cultured alone or with RAW264.7 were seeded in a 96-well plate. Cell number ratio was 3:1 with both groups totaling 8000 cells/well (monoculture, 4T1-Luc cells: 8000 cells; coculture, 4T1-Luc cells: 6000 cells and RAW264.7 cells: 2000 cells). After 24 h of incubation, the cells were treated with RSL3 (200 nM) and then incubated for 24 h. Images are representative of 4T1-Luc/AcGFP cells. Hoechst 33342 was used for staining nuclei. Scale bars: 200 µm. This experiment was repeated twice with similar results. **B** After 24 h of incubation, the cells were treated with RSL3 at the indicated concentrations for 48 h. Cell viability was determined by measuring the fluorescence intensity of AcGFP. Data are presented as means ± SD (*n* = 5). This experiment was repeated twice with similar results. **C** After 24 h of incubation, the cells were treated with RSL3 (125 nM), Lip-1 (1 µM), or their combination for 48 h. Data are presented as means ± SD (*n* = 5). This experiment was repeated twice with similar results. ****P < 0.0001, ***P < 0.001, **P < 0.01 by two-way ANOVA, followed by Bonferroni’s multiple comparison test. N.T. not treated.

Considering that the interaction between macrophages and cancer cells can affect ferroptosis induction in triple-negative breast cancer cells, we investigated whether direct coculture of 4T1-Luc and RAW264.7 cells could alter ferroptosis sensitivity. To distinguish 4T1-Luc cells from RAW264.7 cells, we established fluorescently labeled (AcGFP) 4T1-Luc cells (4T1-Luc/AcGFP). 4T1-Luc/AcGFP and RAW264.7 cells were cocultured in the same dish, incubated, and then treated with RSL3. After RSL3 treatment, the 4T1-Luc/AcGFP cell area was smaller (Fig. 2A) and the fluorescence signal was significantly lower (Fig. 2B) under the coculture conditions than under the monoculture conditions. This result indicated that RSL3 caused more cell death in the cancer cells cocultured with RAW264.7 cells than in those monocultured. Under the direct coculture conditions, we tested whether the cell death caused by RSL3 was due to ferroptosis by using the ferroptosis inhibitor Lip-1. Results showed that treatment with the combination of RSL3 and Lip-1 significantly decreased cell death caused by RSL3 treatment under both monoculture and coculture conditions (Fig. 2C). Collectively, these results suggest that in addition to indirect coculture, direct coculture of cancer cells and macrophages can increase ferroptosis sensitivity.

### Effect of ferroptosis induction caused by ML210, which targets GPx4

**Fig. 3:**
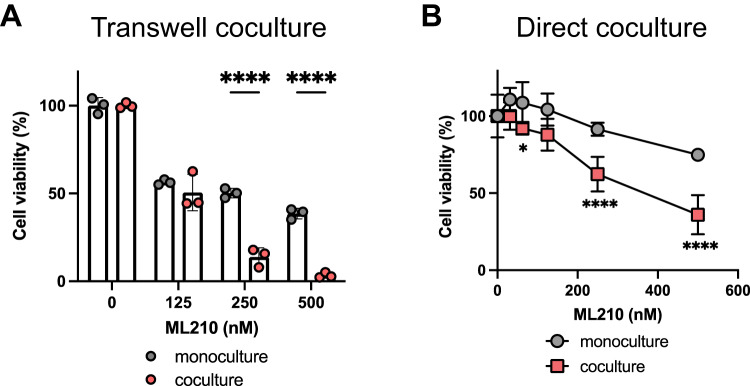
Effect of ferroptosis induction caused by ML210, which targets GPx4. **A** 4T1-Luc cells (5000 cells/well) were cultured alone or with RAW264.7 (5000 cells/well) cells in a 24-well plate and transwell insert with 0.4 µm pore size. After 72 h of incubation, the cells were treated with ML210 at the indicated concentrations for 16 h. Cell viability was measured using the MTT assay. Data are presented as means ± SD (*n* = 3). This experiment was repeated twice with similar results. **B** 4T1-Luc/AcGFP alone or with RAW264.7 cells were seeded in a 96-well plate. Cell number ratio was 3:1 (total 8000 cells/well). After 24 h of incubation, the cells were treated with ML210 at the indicated concentrations for 48 h. Cell viability was determined by measuring the fluorescence intensity of AcGFP. This experiment was repeated twice with similar results. Data are expressed as means ± SD (*n* = 5). ****P < 0.0001, *P < 0.05 by two-way ANOVA, followed by Bonferroni’s multiple comparison test.

RSL3 induces ferroptosis by inhibiting GPx4 [16]. To further investigate the effect of the coculture on ferroptosis induction, we evaluated the effect of ML210, another GPx4 inhibitor, on ferroptosis sensitivity in the coculture of 4T1-Luc and RAW264.7 cells. After ML210 treatment, 4T1-Luc cell viability was lower under the indirect (Fig. 3A) and direct (Fig. 3B) coculture conditions than under the monoculture conditions (Fig. 3A, B). These results suggest that targeting GPx4 can enhance ferroptosis in coculture with macrophages. We also examined whether ferroptosis induction by targeting systems other than GPx4 has similar effects. In addition to the inhibition of GPx4 inhibition, the inhibition of system xc-, which comprises xCT (SLC7A11) and 4F2 heavy chain (SLC3A2), which are known amino acid transporters in the plasma membrane, also causes ferroptosis [17]. To assess the effect of ferroptosis induction by targeting system xc-, we utilized erastin as an inhibitor and tested it under the conditioned medium-treated, indirect coculture, and direct coculture conditions. The sensitivity to erastin was similar to that of the control group when 4T1-Luc cells were incubated with the conditioned medium from RAW264.7 cells (Supplementary Fig. 2A). The rate of ferroptosis induced by erastin treatment significantly increased under the indirect coculture conditions, similar to GPx4 inhibition (Supplementary Fig. 2B). However, under the direct coculture conditions, the rate of ferroptosis induced by erastin decreased (Supplementary Fig. 2C). We further tested the effect of IKE, another compound targeting system xc-, and found that it similarly decreased and increased cell viability under the indirect and direct coculture conditions, respectively (Supplementary Fig. 2D, E). These results suggest that ferroptosis sensitivity increases under indirect coculture conditions but changes under direct coculture conditions depending on the form of ferroptosis induction.

### Intracellular lipid peroxidation increased under coculture conditions

**Fig. 4:**
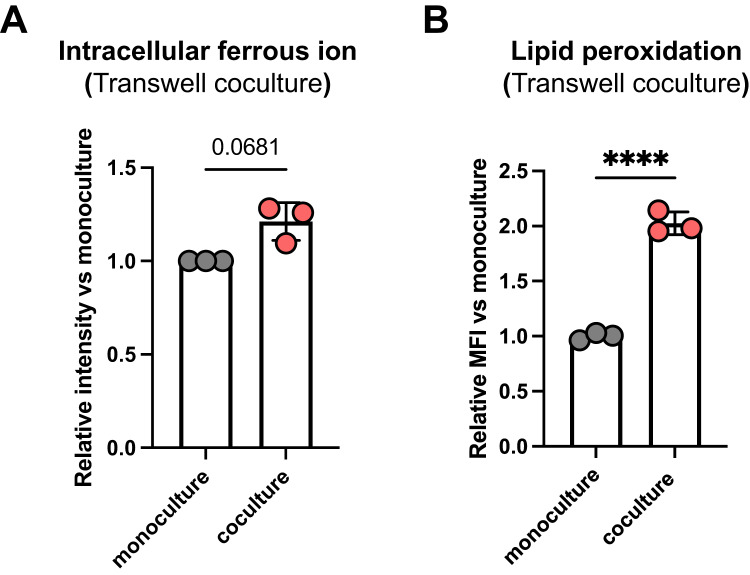
Intracellular ferrous ion and lipid peroxidation in the transwell coculture system. **A** 4T1-Luc cells (5000 cells/well) were cultured alone or with RAW264.7 (5000 cells/well) cells in a 24-well plate and transwell insert with 0.4 µm pore size. After 72 h of incubation, 4T1-Luc cells were stained with FerroOrange, and fluorescence intensity was measured on a plate reader. Data are expressed as means ± SD of three independent experiments. *P* = 0.0681 by two-tailed paired t-test. **B** 4T1-Luc cells (5000 cells/well) were cultured alone or with RAW264.7 (5000 cells/well) cells in a 24-well plate and transwell insert with 0.4 µm pore size. After 72 h of incubation, 4T1-Luc cells were stained with BODIPY 589/591 c11. Then, the collected cells were analyzed using a flow cytometer. Data are expressed as means ± SD (*n* = 3). ****P < 0.0001 by two-tailed unpaired t-test. This experiment was repeated twice with similar results.

Ferroptosis is caused by the accumulation of lipid peroxidation generated by Fenton’s reaction with ferrous ions [18]. Intracellular iron metabolism is also associated with ferroptosis, and macrophages can alter intracellular ion components [19]. Therefore, we assessed the effect of coculture with macrophages on the amount of intracellular ferrous ions in cancer cells. After 72 h of indirect coculture, the amount of ferrous ions was measured using the FerroOrange probe. Results showed that the amount of ferrous ions was higher under coculture conditions than under monoculture conditions (Fig. 4A). Intracellular lipid peroxidation was then evaluated under monoculture and coculture conditions using the indirect coculture method. Using the BODIPY 589/591 C11 probe, we detected lipid peroxidation under the indirect coculture conditions. Flow cytometric analysis showed that lipid peroxidation significantly increased under coculture conditions (Fig. 4B). These data suggest that lipid peroxidation partially increases with the accumulation of intracellular ferrous ions and contributes to ferroptosis sensitivity under coculture conditions.

## Discussion

Several molecular targeted drugs have been approved for cancer treatment. However, refractory cancers require drugs that specifically target cancer cells. Thus, novel therapeutic options need to be developed urgently to treat these cancers. Despite several attempts to overcome these refractory cancers, novel drugs that can eliminate these cancers are still needed. Almost all molecular targeted drugs aim to induce apoptosis in cancer cells, and novel types of agents that specifically induce cancer cell death have attracted increasing attention. Thus, interest in ferroptosis as a therapeutic option for cancer has grown.

The present study demonstrated that the ferroptosis induced by the GPx4 inhibitors RSL3 and ML210 increased when the cancer cells were co-incubated with macrophages (Figs. 1–3). In the viewpoint of secretion, long noncoding-ENDOG-1:1 from exosomes suppresses ferroptosis in gastric cancer stem cells [20]. In addition, immunotherapy-activated CD8 T cells induce ferroptosis in cancer cells via interferon gamma (IFNγ) and IFNγ receptor I axis [21]. Considering that we observed increased ferroptosis sensitivity in the transwell coculture model and direct coculture model, but not in conditioned medium from macrophages (Fig. 1A and Supplementary Fig. 2A), we hypothesized that the continuous and bi-directional interactions between macrophages and cancer cells, facilitated through secretion or direct cell-to-cell contact, could potentially influence the susceptibility of cancer cells to ferroptosis. At this point, investigating secreted proteins from cancer and macrophage cells, as well as direct interactions that increase the ferroptosis susceptibility of cells, warrants further exploration.

In this study, we utilized 4T1-Luc cells as a model cancer cell line for triple-negative breast cancer and RAW264.7 cells as a model of macrophage cells. Considering the heterogeneous pathology of triple-negative breast cancer [22], further investigation using several human triple-negative breast cancer cell lines is warranted. Additionally, to thoroughly investigate the effect of coculture with macrophage cells and their phagocytic function, the primary cell culture method is also needed as part of future work. As an application of our findings to clinical setting, in addition to in vivo work, macrophage–cancer cell interaction on clinical tissue sample should be explored using spatial transcriptomic analysis [23].

In this study, although further imaging analysis using fluorescence microscopy is needed, lipid peroxidation increased in the transwell coculture system (Fig. 4B), suggesting that macrophage–cancer cell interactions promoted lipid peroxidation. Thus, the interaction via secretion may alter the signaling pathway to enhance lipid peroxidation in cancer cells. Lipid peroxidation, including that mediated by Fenton’s reaction, is a complex and diverse process regulated by various cellular systems. The relatively weaker increase in intracellular ferrous ion amounts compared with lipid peroxidation (Fig. 4A, B) implies that pathways beyond the Fenton reaction-mediated lipid peroxidation are activated to regulate lipid peroxidation. Polyunsaturated fatty acids are crucial for lipid peroxidation, and their biosynthesis determines the ferroptosis sensitivity of gastric cancer cells [24]. Additionally, GPx4 and ferroptosis suppressor protein 1 independently inhibit lipid peroxidation and ferroptosis [25]. Therefore, further investigation is required to elucidate the mechanisms by which macrophage–cancer cell interactions enhance lipid peroxidation and identify the pathway dominating this mechanism.

Our findings demonstrate that GPx4 inhibitors, such as RSL3 and ML210, indirectly and directly induce ferroptosis in cancer cells cocultured with macrophages (Figs. 1–3). Meanwhile, inhibition of system xc- using erastin and IKE promoted ferroptosis in the indirect coculture system but not in the direct coculture system (Supplementary Fig. 2). This distinct response can be ascribed to the specific coculture system involving cancer cells and macrophages. Previous studies reported that macrophage and cancer cell distances [26] and macrophage infiltration [27] vary in the TME, indicating several ways in which macrophages can influence tumor cells, either directly or indirectly. In addition, compared to GP4x inhibitors such as RSL3, erastin and IKE are targeting system xc- in the plasma membrane. To address the opposite effect of GPx4 inhibitor and system xc- inhibition in the direct coculture system (Fig. 2 and Supplementary Fig. 2C, D), localization of and expression of targeted proteins are needed to be explored. Collectively, the further work might clarify the localization of macrophage in the TME and its crucial role in altering the ferroptosis signaling pathway and determining sensitivity to ferroptosis inducers, such as RSL3 and erastin.

The results of the present study suggest that macrophage–cancer cell interactions significantly affect ferroptosis susceptibility. Reliable biomarkers of efficacy must be identified to employ ferroptosis-inducing compounds in cancer treatment. Our findings offer critical insights into the importance of macrophages in the TME as determinants of the ferroptosis response.

## Materials and methods

### Cell culture

4T1-Luc cells were purchased from the Japanese Collection of Research Bioresources (JCRB; Osaka, Japan, JCRB1447), and RAW264.7 cells were obtained from the American Type Culture Collection (ATCC; Manassas, VA, USA). 4T1-Luc cells were cultured in RPMI-1640 (FUJIFILM Wako Pure Chemical, Osaka, Japan) containing 10% fetal bovine serum (FBS; Biosera, Nuaille, France) and 1% (v/v) penicillin–streptomycin-amphotericin B suspension (FUJIFILM Wako Pure Chemical). RAW264.7 cells were cultured in Dulbecco’s modified Eagle’s medium (DMEM; FUJIFILM Wako Pure Chemical) containing 10% FBS (Biosera) and 1% (v/v) penicillin–streptomycin–amphotericin B suspension (FUJIFILM Wako Pure Chemical). Both cell lines were maintained at 37 °C under 95% air and 5% CO_2_ atmosphere. All experiments were performed using cells of less than 20 passages. Regular assessments for *Mycoplasma* contamination were performed using commercially available kits (EZ-PCR™ Mycoplasma Detection Kit, Biological Industries, Beit Haemek, Israel).

### Reagents

RSL3, ML210, and liproxstatin-1 (Lip-1) were purchased from Selleck Chem (Houston, TX, USA). Deferoxamine (DFO) was purchased from Sigma-Aldrich (St. Louis, MO, USA). Erastin and imidazole ketone erastin (IKE) were purchased from MedChem Express (Monmouth Junction, NJ, USA).

### Preparation of conditioned medium

RAW264.7 cells were seeded in a 6-well plate at a density of 2.0 × 10^5^ cells/well. After 24 h of incubation, the cells were washed with phosphate-buffered saline (PBS) and then incubated with DMEM containing 1% FBS for 24 h. The conditioned medium was collected by centrifugation of the supernatant at 300 × *g* for 5 min at 4 °C to remove cell debris. FBS concentration in the conditioned medium from RAW264.7 cells was adjusted to 10% by adding FBS. Subsequently, the conditioned medium (containing 10% FBS) from RAW264.7 cells was mixed with 10% FBS DMEM at a ratio of 1:4. As a control, unconditioned 10% FBS DMEM was used.

### Indirect coculture system with transwell chamber

4T1-Luc and RAW264.7 cells were seeded in a 24-well plate (CORNING, Corning, NY, USA) and transwell inserts with 0.4 µm pore size, respectively, and then cocultured for 72 h. We used DMEM in this system after confirming that the growth rate of 4 T1-Luc cells was not considerably different from that of the cells grown in RPMI-1640 (data not shown).

### Direct coculture system

To coculture 4T1-Luc and RAW264.7 cells directly in the same dish, we established AcGFP-labeled 4T-Luc cells (4T1-Luc/AcGFP) through lentiviral transduction. To produce the lentivirus, the pLVSIN-AcGFP-C1 vector (Takara Bio, Shiga, Japan) with psPAX2 and pMD2.G (Addgene, Watertown, MA, USA) was transfected into HEK293T cells (ATCC) using Fugene HD (Promega, Madison, WI, USA) following the manufacturer’s instructions. After 48 h, the lentivirus was collected, and cell debris was excluded using a 0.45 µm filter (Millipore, Burlington, MA, USA). We infected 4T1-Luc cells with the lentivirus supernatant in the presence of 8 µg/mL Polybrene (Millipore) for 48 h and then obtained AcGFP-positive clones through limiting dilution. Subsequently, 4T1-Luc/AcGFP and RAW264.7 cells were mixed and seeded in a 96-well plate. Cell number ratio was 3:1 with both groups totaling 8000 cells/well (monoculture, 4T1-Luc cells: 8000 cells; coculture, 4T1-Luc cells: 6000 cells and RAW264.7 cells: 2000 cells). After 24 h of incubation, the indicated reagent was treated and cell viability was measured. For imaging analysis, Hoechst 33342 (Nacalai tesque, Kyoto, Japan) was used for staining nuclei.

### Cell viability test

The viability of 4T1-Luc cells was assessed using different assays dependent on culture conditions.

For the 3-(4,5-dimethyl-thiazol-2-yl)-2,5-diphenyl tetrazolium bromide (MTT; Sigma-Aldrich) assay, cells were seeded in a 96-well plate (Thermo Fisher Scientific, Waltham, MA, USA) at a density of 5000 cells/well and then treated with the indicated reagents for the specified time, which varied in each experiment. After incubation, the cells were added with 0.5 mg/mL MTT solution and then cultured for 3 h. Formazan crystals were dissolved by adding dimethyl sulfoxide, and the absorbance was obtained at 570 nm. Cell viability was normalized to that of the untreated group. After indirect coculture for 72 h, the cells were treated with the indicated reagents for an additional 16 h. After treatment, cell viability was measured using the MTT assay as described above.

In the indirect coculture system, cell viability was measured through crystal violet staining. After coculture for 72 h, the cells were treated with the indicated reagents for an additional 16 h, fixed with 4% paraformaldehyde (FUJIFILM Wako Pure Chemical), and then stained with crystal violet (FUJIFILM Wako Pure Chemical). Images of stained cells were acquired using a BZ-X800 microscope (Keyence, Osaka, Japan). The stained area in each image was quantified using ImageJ (ver.1.53q, National Institutes of Health, Bethesda, MD, USA). The stained cells were measured by dissolving crystal violet in 50% ethanol (FUJIFILM Wako Pure Chemical) and obtaining the absorbance at 570 nm. Cell viability was normalized to that of the untreated group.

In the direct coculture system, cell viability was evaluated by measuring the fluorescence intensity of 4T1-Luc/AcGFP cells on a plate reader (Infinite M1000; TECAN, Männedorf, Switzerland) with excitation at 475 nm and emission at 505 nm. Cell viability was normalized to that of the untreated group. Fluorescent-positive cell images were acquired using CellVoyager CV8000 (CV8000; Yokogawa, Tokyo, Japan) to capture the viability of 4T1-Luc/AcGFP cells.

### Ferrous ion quantification

After indirect coculture, the cells were washed with PBS and incubated with FerroOrange (DOJINDO, Kumamoto, Japan) at a final concentration of 1 µM for 30 min, and fluorescence intensity was measured on a plate reader (Infinite M1000, TECAN, Zurich, Switzerland) at 543 nm excitation and 580 nm emission.

### Lipid peroxide quantification

After indirect coculture, the cells were treated with the indicated reagents for 2 h. After being washed with PBS, the cells were incubated with 5 µM BODIPY 589/591 C11 (Thermo Fisher Scientific) for 1 h, trypsinized, and then analyzed using a MACSQuant X flow cytometer (Miltenyi Biotec, Bergisch Gladbach, Germany).

### Statistical analysis

Statistical analyses were performed using Prism 9 for MacOS (GraphPad Software, San Francisco, CA, USA). Data are presented as means ± standard deviation (SD). *P*-values were calculated using two-tailed t-test, and one-way or two-way analysis of variance followed by either Bonferroni’s or Tukey’s post-hoc test. Statistical significance was considered at *P* < 0.05.

### Reporting summary

Further information on research design is available in the Nature Research Reporting Summary linked to this article.

### Supplementary information

Supplementary Figures

Reporting Summary

## Data Availability

All data are included in this published article and its supplementary files.
