# Supplementary material for: Coculture with macrophages alters ferroptosis susceptibility of triple-negative cancer cells
Source: Cell Death Discov. 2024 Mar 1;10:108. doi: 10.1038/s41420-024-01884-w (PMC10907599; doi:10.1038/s41420-024-01884-w)
Supplement: Supplementary file 1 — Supplementary Figures [file 41420_2024_1884_MOESM1_ESM.pptx]

## Slide 1
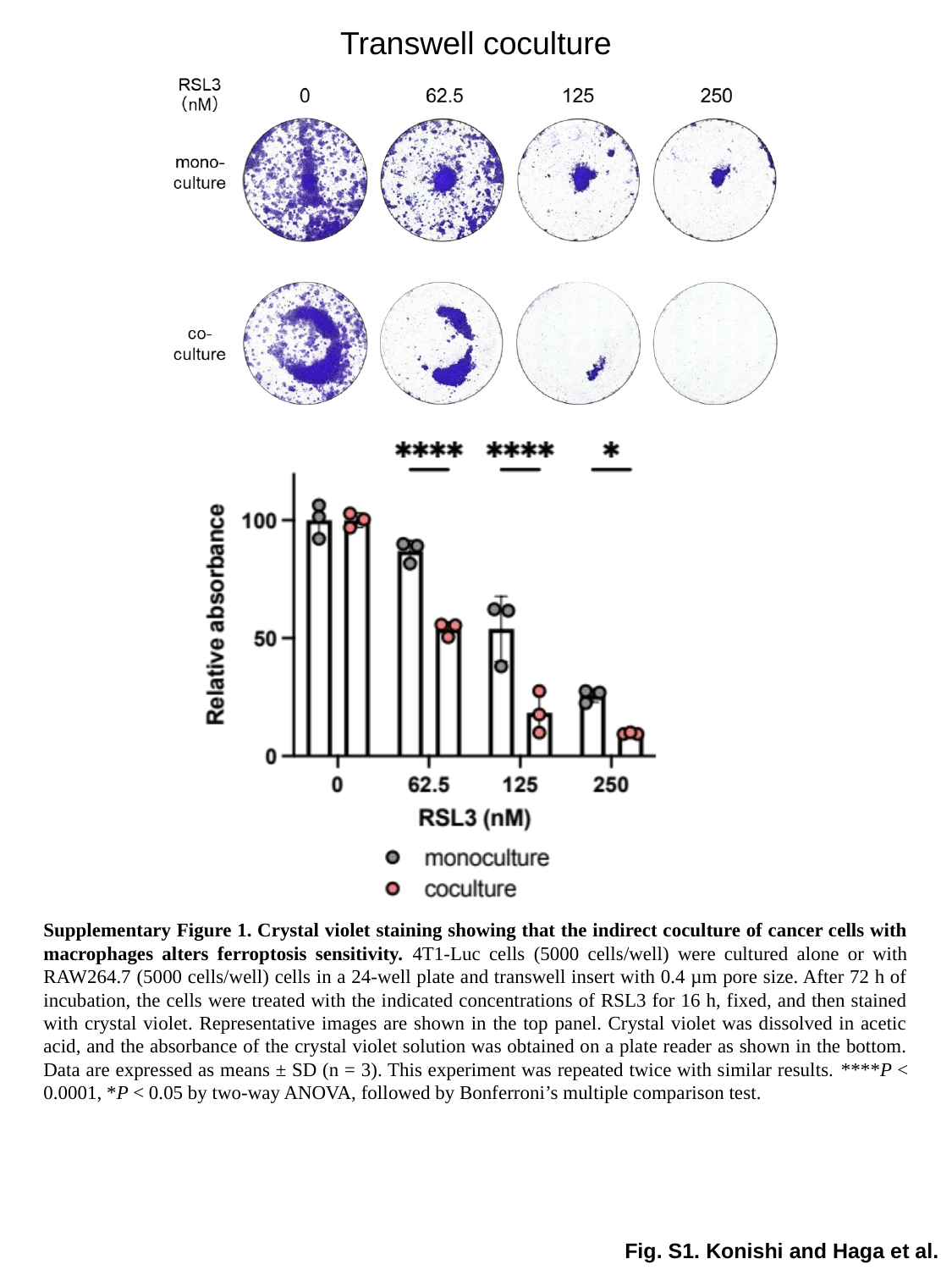

Transwell coculture
Supplementary Figure 1. Crystal violet staining showing that the indirect coculture of cancer cells with macrophages alters ferroptosis sensitivity. 4T1-Luc cells (5000 cells/well) were cultured alone or with RAW264.7 (5000 cells/well) cells in a 24-well plate and transwell insert with 0.4 µm pore size. After 72 h of incubation, the cells were treated with the indicated concentrations of RSL3 for 16 h, fixed, and then stained with crystal violet. Representative images are shown in the top panel. Crystal violet was dissolved in acetic acid, and the absorbance of the crystal violet solution was obtained on a plate reader as shown in the bottom. Data are expressed as means ± SD (n = 3). This experiment was repeated twice with similar results. ****P < 0.0001, *P < 0.05 by two-way ANOVA, followed by Bonferroni’s multiple comparison test.
Fig. S1. Konishi and Haga et al.

## Slide 2
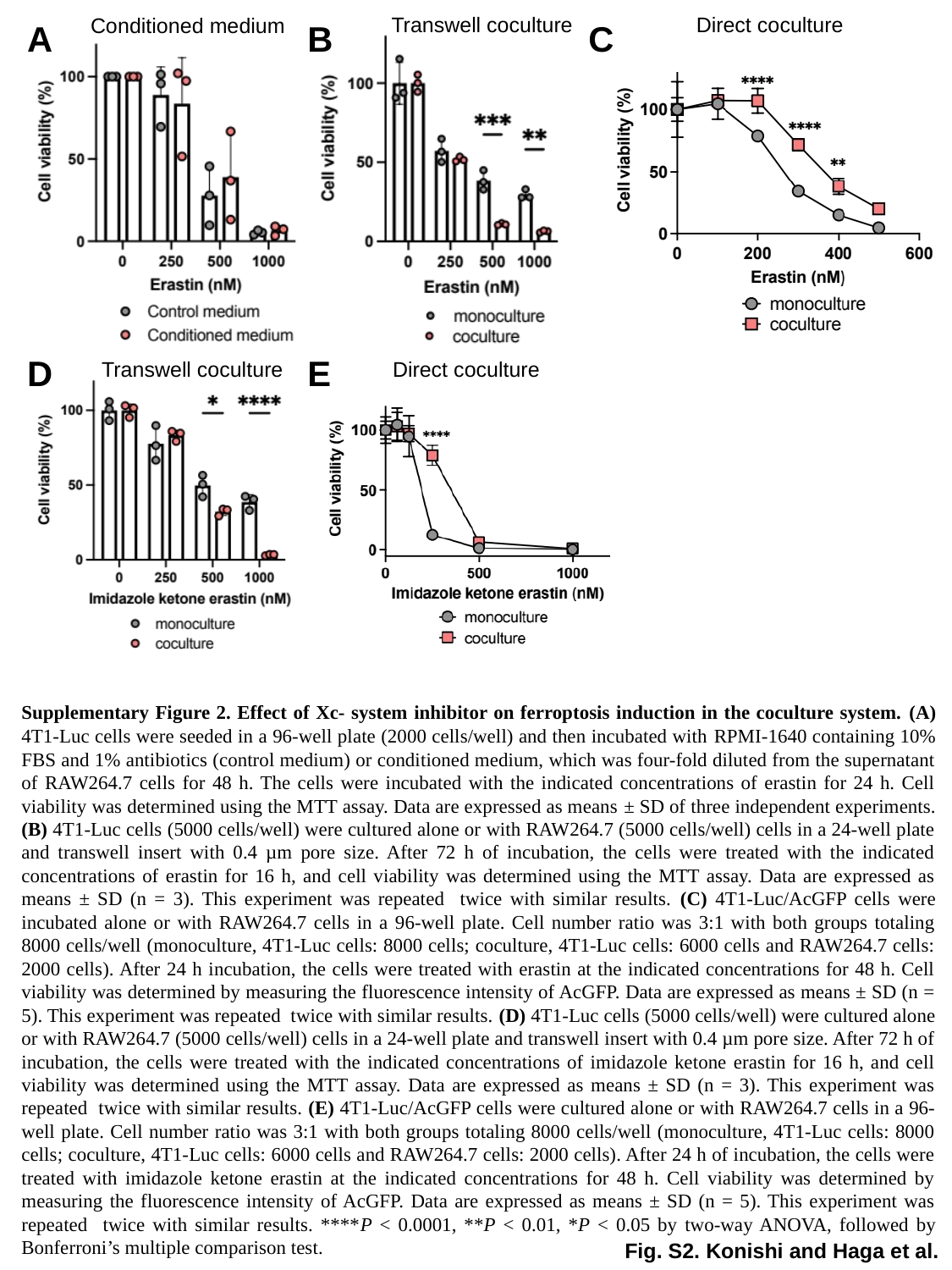

Transwell coculture
Direct coculture
Conditioned medium
A
B
C
D
E
Transwell coculture
Direct coculture
Supplementary Figure 2. Effect of Xc- system inhibitor on ferroptosis induction in the coculture system. (A) 4T1-Luc cells were seeded in a 96-well plate (2000 cells/well) and then incubated with RPMI-1640 containing 10% FBS and 1% antibiotics (control medium) or conditioned medium, which was four-fold diluted from the supernatant of RAW264.7 cells for 48 h. The cells were incubated with the indicated concentrations of erastin for 24 h. Cell viability was determined using the MTT assay. Data are expressed as means ± SD of three independent experiments. (B) 4T1-Luc cells (5000 cells/well) were cultured alone or with RAW264.7 (5000 cells/well) cells in a 24-well plate and transwell insert with 0.4 µm pore size. After 72 h of incubation, the cells were treated with the indicated concentrations of erastin for 16 h, and cell viability was determined using the MTT assay. Data are expressed as means ± SD (n = 3). This experiment was repeated twice with similar results. (C) 4T1-Luc/AcGFP cells were incubated alone or with RAW264.7 cells in a 96-well plate. Cell number ratio was 3:1 with both groups totaling 8000 cells/well (monoculture, 4T1-Luc cells: 8000 cells; coculture, 4T1-Luc cells: 6000 cells and RAW264.7 cells: 2000 cells). After 24 h incubation, the cells were treated with erastin at the indicated concentrations for 48 h. Cell viability was determined by measuring the fluorescence intensity of AcGFP. Data are expressed as means ± SD (n = 5). This experiment was repeated twice with similar results. (D) 4T1-Luc cells (5000 cells/well) were cultured alone or with RAW264.7 (5000 cells/well) cells in a 24-well plate and transwell insert with 0.4 µm pore size. After 72 h of incubation, the cells were treated with the indicated concentrations of imidazole ketone erastin for 16 h, and cell viability was determined using the MTT assay. Data are expressed as means ± SD (n = 3). This experiment was repeated twice with similar results. (E) 4T1-Luc/AcGFP cells were cultured alone or with RAW264.7 cells in a 96-well plate. Cell number ratio was 3:1 with both groups totaling 8000 cells/well (monoculture, 4T1-Luc cells: 8000 cells; coculture, 4T1-Luc cells: 6000 cells and RAW264.7 cells: 2000 cells). After 24 h of incubation, the cells were treated with imidazole ketone erastin at the indicated concentrations for 48 h. Cell viability was determined by measuring the fluorescence intensity of AcGFP. Data are expressed as means ± SD (n = 5). This experiment was repeated twice with similar results. ****P < 0.0001, **P < 0.01, *P < 0.05 by two-way ANOVA, followed by Bonferroni’s multiple comparison test.
Fig. S2. Konishi and Haga et al.
